# To Pee, or Not to Pee: A Review on Envenomation and Treatment in European Jellyfish Species

**DOI:** 10.3390/md14070127

**Published:** 2016-07-08

**Authors:** Louise Montgomery, Jan Seys, Jan Mees

**Affiliations:** 1 Flanders Marine Institute, InnovOcean Site, Wandelaarkaai 7, Ostende 8400, Belgium; jan.seys@vliz.be (J.S.); jan.mees@vliz.be (J.M.); 2 College of Medical, Veterinary & Life Sciences, Graham Kerr Building, University of Glasgow, Glasgow G12 8QQ, UK; 3 Ghent University, Marine Biology Research Group, Krijgslaan 281, Campus Sterre-S8, Ghent B-9000, Belgium

**Keywords:** jellyfish, European, cnidarians, nematocyst, stings, envenomation, pain, treatment, relief

## Abstract

There is a growing cause for concern on envenoming European species because of jellyfish blooms, climate change and globalization displacing species. Treatment of envenomation involves the prevention of further nematocyst release and relieving local and systemic symptoms. Many anecdotal treatments are available but species-specific first aid response is essential for effective treatment. However, species identification is difficult in most cases. There is evidence that oral analgesics, seawater, baking soda slurry and 42–45 °C hot water are effective against nematocyst inhibition and giving pain relief. The application of topical vinegar for 30 s is effective on stings of specific species. Treatments, which produce osmotic or pressure changes can exacerbate the initial sting and aggravate symptoms, common among many anecdotal treatments. Most available therapies are based on weak evidence and thus it is strongly recommended that randomized clinical trials are undertaken. We recommend a vital increase in directed research on the effect of environmental factors on envenoming mechanisms and to establish a species-specific treatment. Adequate signage on jellyfish stings and standardized first aid protocols with emphasis on protective equipment and avoidance of jellyfish to minimize cases should be implemented in areas at risk.

## 1. Introduction

Over the past few decades, jellyfish blooms have caused a great deal of disturbance across the world with the perception that their increasing numbers are damaging fishing gear, blocking power plants and impacting on fragile fish stocks. This may be due to climatic changes, overfishing, eutrophication and habitat alteration [1,2,3,4,5]. Due to this increase, jellyfish envenomation has become an increasing problem not only for public health, tourists, and fishermen, but also indirect impact on the economy [6]. Globally, approximately 10,000 species make up the Cnidarian *phylum*, with 1% recognized as a threat to human health. Within the Cnidarian *phylum* most health problems arise from the representatives of scyphozoans (true jellyfish), cubozoans (box jellyfish) and hydrozoans (siphonophores and hydroids) [7,8,9,10].

Jellyfish stings have become a familiar summer hazard in Europe with an increase in marine activities taking place at this time of year [11]. It is thought that as many as 150 million jellyfish stings take place annually worldwide emphasizing the need for more conclusive research on this topic [7,12]. Envenomation mainly causes cutaneous reactions such as pain and itching, however, systemic symptoms including cardiac and neurological complications can result from venom entering the circulatory system. Such serious reactions, which can occasionally be fatal, are most commonly consequences of stings inflicted by venomous jellyfish such as the Australian species *Chironex fleckeri*. However death, although rare, can occur from species that are found in Europe, such as from *Cyanea capillata* and *Physalia physalis* associated with an allergic response or aggravating prior health concerns [10,13,14].

Although jellyfish seem relatively simple and have existed since the Pre-Cambrian era, they are sophisticated organisms with complex life cycles and advanced envenoming mechanisms [1,15]. Little is known about the factors that affect their lifecycles with regard to the true composition of their toxins and how to treat their stings. There is much confusion among the public on the first aid protocol for jellyfish envenomation, which is further complicated by the portrayal of quick treatments such as urine and meat tenderizer as suitable therapeutic agents from extrapolated results without discussing the limitations of the findings [16,17]. There is a great deal of literature on the treatment of jellyfish envenomation, which is contradictive, uncertain and unjustified, and thus reflects knowledge at this time [15,17]. With no universal therapeutic available, there is a need to develop species or genus specific therapies, as the nature of the venom is organism dependent [13]. Individuals can react differently to the venom depending on their health, where they are stung both anatomically and geographically, the surface area of the sting site, and the rate of peripheral circulation, which may be dependent on sex, age and size [15,18,19]. Anecdotal treatment is accepted due to the scarcity of statistically significant findings of evidence-based medicine (EBM). However, many currently accepted anecdotal treatments can exacerbate symptoms through further nematocyst release [15,16,17].

We reviewed first aid treatments and health problems associated with European jellyfish envenomation and compiled the most reliable treatments available for stings of the 41 European species. The envenomation process and an accord of the most reliable treatments for general and common envenoming species are provided, as well as recommendations for further research.

## 2. Methods

79 publications on European jellyfish species envenomation, toxicology and treatment were identified through Web of Knowledge (WoK) [20]. The term “jellyfish” was defined as all European scyphozoan and cubozoan species, and frequent envenoming hydrozoan species. All European scyphozoan and cubozoan species were identified with the aid of the World Register of Marine Species (WoRMS) [21] and based upon a publication search that stated species found in European waters that were not recorded in WoRMS. Hydrozoan species were chosen through the identification of recurring species in envenomation cases on WoK, and with reference to hydrozoan species selected by MED-jellyrisk, an integrated monitoring program of jellyfish outbreaks in the Mediterranean Sea [22]. Forty one species were considered: all 37 scyphozoans, the single cubozoan, and three hydrozoan species.

The following terms were searched: “jellyfish AND envenomation AND Europe”; “jellyfish AND toxicology AND Europe”; “jellyfish AND envenomation”; “jellyfish AND toxicology AND * *species name”* (* *out of the 41 species identified in Europe*); *“*jellyfish AND sting AND treatment”; “* *species name*”*.* The papers were selected after skim reading the document. The full papers were then examined and the papers selected met at least one of the following criteria: (a) the publication was related to European jellyfish; (b) the publication was about species in other continental waters but is also regarded as a European species; (c) the paper discussed innovative first aid treatments on international jellyfish envenomation; (d) the paper referred to species of the same genus as a European species. Further relevant articles were also selected from referenced papers due to a lack of papers available from the WoK search.

Publications on laboratory studies, field studies, case studies with randomized and nonrandomized controlled trials, letters to the editor, observational case studies with and without controls, literature reviews, books and expert opinions were all used. Some publications presented individual case studies while others presented larger scale investigations. Some had poor experimental protocol suggesting why anecdotal treatments are greatly accepted.

The relevant information on jellyfish species: markings induced cutaneous symptoms and systemic symptoms suffered upon immediate envenomation as well as long-term medical problems were extracted. Associated treatments were recorded with the effect of the treatment noted on whether symptoms improved, had no effect or exacerbate the sting and related symptoms. Cross-reactive species, the reaction between two different species, were also documented.

## 3. The Organisms

### 3.1. Anatomy and Physiology of the Stinging Mechanism

**Figure 1:**
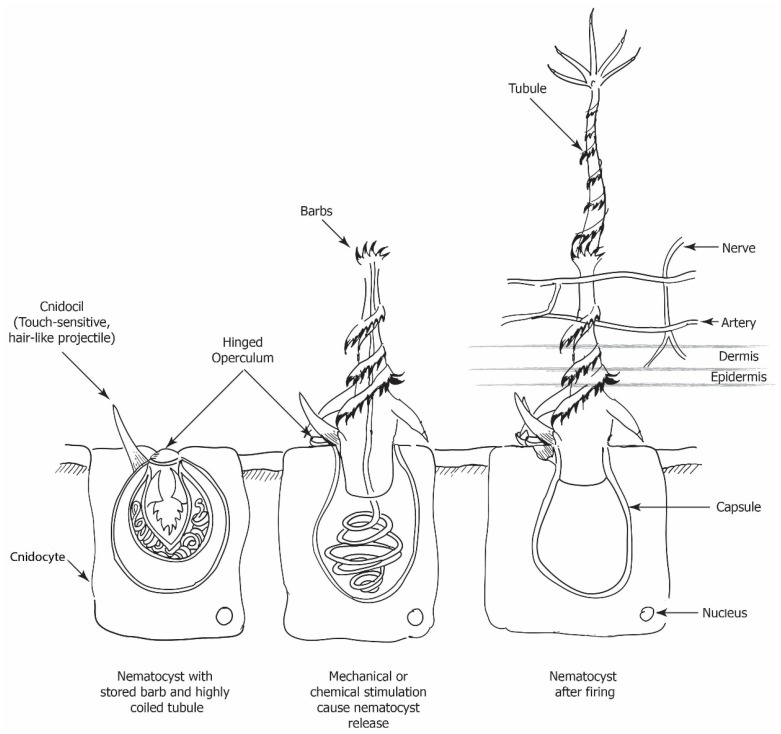
Nematocyst structure and mechanism.

Cnidarians have epithelial stinging cells (Figure 1), called cnidocytes, which house cnidocysts (also known as cnidae) and are clustered into batteries along the tentacles and in some species on the bell. Nematocysts are one of three categories of cnidocysts found in cnidaria. Nematocysts are enclosed within cnidocytes. Jellyfish tentacles contain these specialized nematocyst structures, which allow envenomation due to highly coiled tubules armed with spines and is species dependent [23,24,25,26].

There are several morphological types of nematocysts with a variation of discharge velocities, capsular size, tubule length, and trajectory. Variable reported stinging potencies of individual nematocyst types have been reported that suggest the severity of the sting differing between species in different geographic locations, dependent on nematocyst composition [19,24,27,28].

### 3.2. Factors Involved during Envenomation

The spring loaded barbed tubules are contained within capsules which fire upon mechanical or chemical stimulation [24,26,29]. The nematocyst tubule is released from the capsule upon stimulation within a fraction of a second (700 ns), in what is thought to be one of the fastest mechanisms present in nature at 18.6 m·s^−1^ [26]. Once the tubule is everted after the uncoiling of the thread, the victims epidermis along with upper dermis is penetrated and venom is injected from the capsule. This may result in fatal, systemic, or local cutaneous reactions [30]. Tubule length is species dependent, up to 850 µm long, and has been found to play a role in whether a species is harmless or not to humans [10,19,23,31]. Venom injection occurs along the full length of the tubule, allowing deposition of venom over a maximal surface area of the vascular bed. This affects the integrity of the microvasculature, leading to inflammation and the further entrance of venomous components into the circulatory system [10].

Species tend to be non-venomous to humans either due to the inadequate length of the nematocyst shaft to penetrate deep enough into the dermis to produce a toxic effect, or because some toxins may not cause a reaction in humans, but are merely potent enough for prey and protection against predators. Nematocysts can be in the “ready fire” position or hidden and withdrawn by a fibrillar network to an unexposed position [18].

Not all tentacle contact results in a sting, defined as immediate pain plus a rash, as Burnett found there to be a “miss” a third of the time when tentacles came into contact with the skin [16]. Jellyfish tentacles hold between a few thousand to several billion nematocysts [7,25]. Nematocysts structures are still able to fire when separated from the organism or when the organism is dead [18]. Dried nematocysts, which come in contact with water also can cause envenomation if handled, however, the firing time is decreased [7,10,23].

### 3.3. Venom Constituents and Actions

Very little is known about the toxicity of jellyfish species, with variability found within a single species. *A. aurita* has been found to present a dramatic difference around the world with some areas presenting more severe symptoms than currently found in Europe. Stronger envenomations have been reported by the “New World” *A. aurita* in Australia, Gulf of Mexico and Florida [19].

Jellyfish venoms are composed of a concoction of toxic antigenic polypeptides and pathogenic enzymes to humans, which can lead to local cutaneous reactions, systemic or fatal responses. The active component of the venom is still difficult to determine. Jellyfish composition and difficulties extracting isolated crude venom makes it very challenging to identify the specific components of these venoms. Each venomous molecule has a variation in how it affects physiological processes. The differing factors within venom are believed to target separate organs with distinctive pharmacokinetics, resulting in progressive organ failure, and consequently, the reason why envenomations are challenging to treat [32,33]. Jellyfish are present worldwide with the most harmful species found to occur more commonly in warm tropical waters, but also areas further north, in Europe, particularly in the Mediterranean Sea. Venom has been found to contain hemolysin predominantly in larger pelagic individuals [34,35,36].

The efficacy of the toxin is also dependent on a number of other elements: the type of nematocyst, the penetrating power of the nematocysts needle, molecular size of venom, surface area of the exposed skin, site of bodily injury, body weight, and the victims sensitivity to the venom and dependence on the biological target [34,37,38,39,40].

## 4. Human Pathophysiology after Envenomation

### 4.1. Cutaneous and Systemic Symptoms

No one has been found to be resistant to the pain of jellyfish envenomation. Nonetheless, some people may be more resistant to a low dose of toxins than others. This is not the case for a higher dose of the toxin administered by the envenoming jellyfish. Most sting reactions are usually limited to skin, resulting in pain, swelling and itching, which may lead to the necrosis of skin in more severe stings. However systemic reactions, although far rarer, can occur if there is a large envenomation or allergic reaction such as angioedema or anaphylaxis. Cutaneous marking and the pattern of lesions aid in the identification of the offending species to subsequently ascertain which first aid protocol would be the most beneficial and prevent the increase of symptoms with the wrong post sting method. A catalog of symptoms inflicted by European species provides an aid in the identification of the species and systemic symptoms that may arise from a large venom dose (Table 1) [9,23,28,41].

Table 1 lists the sting severity, species frequency, cutaneous and systemic symptoms found to occur after an envenomation with post sting identification marking, with given geographic location of European jellyfish species.

Pain from the jellyfish stings can be due to the action of endogenous or exogenous chemical mediators, such as the “kinin-like factor” found in venom. The “kinin-like factor” acts on cutaneous sensory nerves and can lead to reactions like urticaria and erythema, which can last for varied durations and is patient and species dependent. This is believed to be a toxic phenomenon due to the appearance upon instant contact and no reported cases of immunity [28]. Jellyfish have chitin, a structural carbohydrate present in tubule spines, which plays a role in triggering the immune response to jellyfish stings. Human genotypes with poor chitinase-like protein have higher rates of diseases, and therefore result in a more severe reaction to stings due to impaired chitin clearance from polymorphisms in chitin-related genes [25,26]. Persistent rubbing of the envenomed area can lead to lichenification, the process by which skin becomes hardened and leathery. Persistent rubbing can also lead to the further discharge of nematocysts directly after the sting [10].

Stinging reactions are more a toxic phenomenon than an allergic reaction and occur without prior exposure, unlike allergic reactions. The toxins produce a similar clinical picture to allergic responses such as hypotension, difficulty breathing and clammy skin [9,28,39]. A toxic lethal action is more common compared to a reaction due to hypersensitivity, and affects the cardiovascular system, respiratory system or kidneys/liver in decreasing order of dosage and increasing time of effect, observed in *Cyanea capillata*, *Carybdea marsupialis and Physalia physalis* [18,32,33,34].

### 4.2. Immune Response

Reaction to the venom can result either directly through the action of the toxin, or indirectly through an immune response, which is humoral (B-cell) and/or cell-mediated (T-cell) [10,26]. Immunological and allergic reactions result from prior exposure to the venom; however do not occur in all people. More serious symptoms of angioedema and anaphylactic shock can result due to a huge release of histamine. This only results due to prior exposure of an envenoming organism with a similar toxic make-up, or distinctly a cross-reactive jellyfish species such as *Pelagia noctiluca* with a *Physalia physalis* sting [19,41]. They are associated with allergic reactions due to the immune system overreacting to antigens. Elevated species-specific immunoglobulins (Ig) can remain in the victim’s bloodstream and can thus lead to an immune response by a subsequent sting of the same species or cross-reacting venom [10,28,47,56,63].

Cross-reactivity between species is due to IgG and IgE-mediated reactions after at least one prior sting leading to sensitization and hence causes a reaction to a subsequent envenomation [10,28,47,56,63]. Elevated species-specific IgG antibodies can persist for several years. These can lead to cross-reactivity between species and can also result in repeated cutaneous eruptions without subsequent stings, which have been found to occur at the original site of the sting [28,64]. Additionally, in rare cases, *Physalia* envenomations have been found to produce lesions distant from the initial sting site [18,28,56,60]. Those with higher specific IgG or IgE antibodies had a higher chance of the development of extra-cutaneous reactions or anaphylactic shock, though this is not universal. Prior exposure is necessary for an allergic response, yet not every subsequent sting can lead to an allergy-like reaction or more severe reaction. There has been no correlation found between the number of jellyfish stings and the height of detectable antibody titer. Conversely, receiving a higher dose of venom during a single sting, which leads to a higher level of IgG titer, increases the threat of anaphylaxis [18,39,47,54].

**Figure 2:**
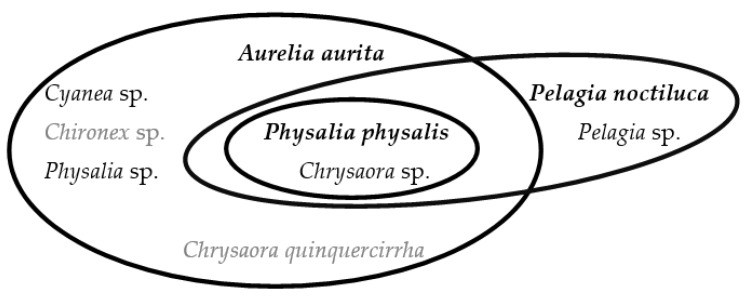
Schematic diagram of cross-reacting jellyfish species mentioned in publications. The species in grey are currently located outside European waters.

Figure 2 is a visual representation of cross-reactive species identified in six publications [10,19,26,34,43,56]. Each oval encompasses the species that have been found to cross-react with the species in bold. The highest species in bold cross-reacts with all lower species in a given oval. A sting from the species highest up in an oval results in a more severe reaction due to the reaction with immunoglobulins (by any species below the initial species in a given oval) already present in the victim, within a certain period of time. For example, an envenomation by *Pelagia noctiluca* can result in an allergic response if antibodies remain in the bloodstream from a previous sting of *Chrysaora* sp*.* It cannot be stated as to whether the species all cross-react every time with each other or what time frame between the stings causes a cross-reaction.

## 5. Treatment

**Figure 3:**
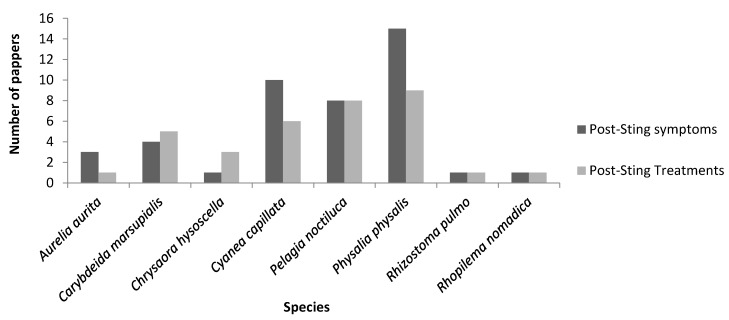
Number of publications on envenomation symptoms and treatments found on European jellyfish species.

Figure 3 visually displays the lack of available publications that discuss the treatments of a very common medical phenomenon. Eight species out of 41 had accessible envenomation literature with many species displaying few papers discussing treatment methods thus portraying the lack of knowledge currently discovered in this literature review.

**Figure 4:**
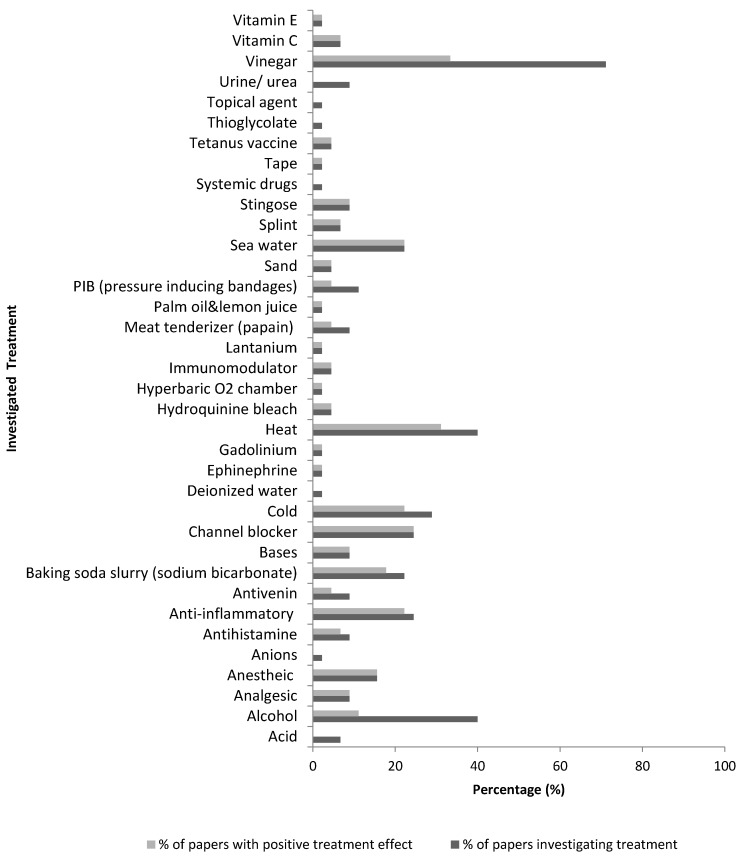
Comparison of the percentage of 63 papers investigating a given treatment, compared to the percentage of papers that concluded beneficial use of the given treatment.

Some treatments can be seen to be more common than others (Figure 4), whilst having little evidence of benefit. The most investigated treatments were seawater (10 papers) and baking soda (10 papers) trialed in 22% of papers, calcium blockers (15 papers) and anti-inflammatories (15 papers) trialed in 24%, cold treatments trialed in 28% (18 papers), alcohol based substance (25 papers) and heat therapies (25 papers) trialed in 40%, and finally vinegar (45 papers) trialed in 71% of papers. Although vinegar was trialed in the majority of publications and a widely used first aid treatment, there was only a positive conclusion in 47% of those publications. Out of the top three investigated studies, only heat treatment presented a significant beneficial treatment of over 50%.

Some of the most popular jellyfish envenomation chemical treatments to provide relief include vinegar (32 papers), alcohol (18 papers) and baking soda slurry (10 papers). Yet, many protocols have been reported to have the reverse effect, with only baking soda found to be beneficial in 80% of investigations. Topical treatment with an anesthetic agent or intravenous (IV) treatments of anesthetics and nerve channel blockers such as benzocaine, lidocaine, and verapamil have been found to provide relief; corticosteroid and antihistamines relieve pain, burning and redness [7,8,13,65]. These have also all shown to inhibit nematocyst release to prevent further venom discharge [66].

**Figure 5:**
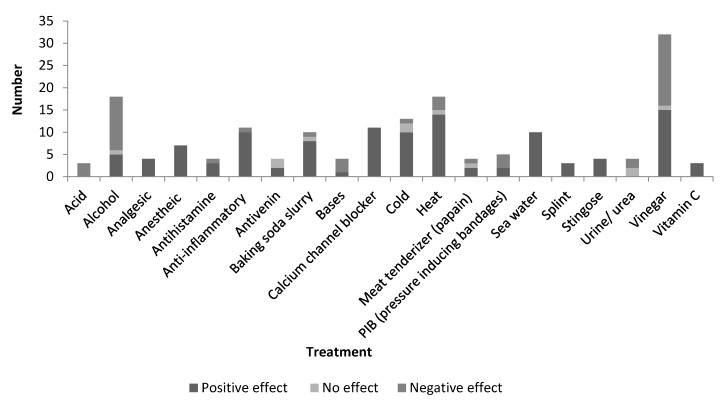
The number of conclusions drawn from reviewed papers displaying beneficial (positive effect), ineffective (no effect), or worsening treatments (negative effect) on the jellyfish sting of treatments found in 5% of papers (3 papers) and above.

Conclusive findings for general treatment of jellyfish stings are displayed in Figure 5. Treatments discussed in less than 5% of papers (below three papers) were removed. Overall the three most commonly used and investigated treatments for post-envenomation treatment are heat treatment, vinegar, and alcohol which are often easily available at the shore. Treatments displaying only positive outcomes are analgesics, anesthetic agents, nerve channel blockers, sea water, a splint, and Stingose (Hamilton Laboratories) which is an Australian aqueous product with 20% aluminium sulphate as the main ingredient. Vinegar has a marginally higher negative effect (16 papers) for sting treatment compared to positive effect (15 papers). Anti-inflammatories, baking soda slurry, and temperature treatment have also been found to portray a greater positive effect than negative.

Table 2 displays the overall effect of treatment used on jellyfish species through color codes, with the number of papers concluding a positive effect (+), a neutral effect, and a negative effect (−) to aid identification of best possible species-specific treatment.

**Figure 6:**
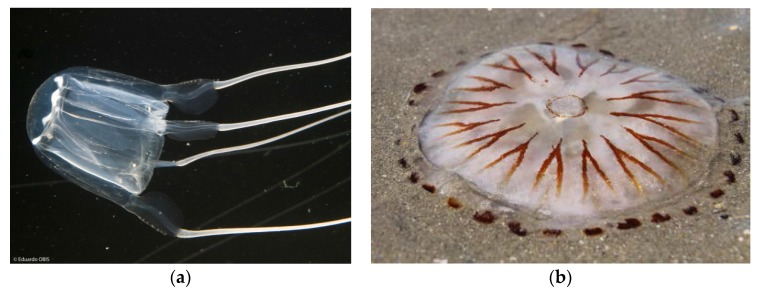
(**a**) Obis, E. *Carybdea marsupialis* are small transparent box jellyfish with a tentacle on each of the four corners. Photograph reproduced with permission from LIFE CUBOMED project (LIFE08 NAT/ES/0064) and the photographer Eduardo Obis [67]; (**b**) de Wulf, L. *Chrysaora hysoscella* is white-yellow with 16 brown paired bands on the bell surface.

On the whole, these are the general recommended first aid treatments for European jellyfish envenomation:

(1) Rescue and life-saving measures to ensure the patient is responsive and stable (Basic Life Support, Epinephrine injection);
(2) Ensure victim stays relaxed and still to prevent venom circulation;
(3) Administer oral analgesic;
(4) Wash tentacles off with seawater, **NOT** freshwater;
(5) **ONLY** for ***Carybdea marsupialis*** (Cubozoa) or ***Chrysaora hysoscella*** (Scyphozoa) shown in Figure 6: Immerse area in vinegar (4%–6% acetic acid) for at least 30 s;
(6) Remove clinging tentacles (if possible, not with bare hands);
(7) Apply baking soda slurry (50% sodium bicarbonate and 50% seawater) for several minutes and rinse off with seawater;
(8) Immerse the affected area in continuous 42–45 °C water for 30 min or till pain is suppressed;
(9) Hospitalization is required if onset of systemic symptoms or intense pain does not subside;
(10) Symptomatic treatment: Antihistamine/Topical steroids/Immunomodulatory drugs.

## 6. Avoidance

The avoidance of jellyfish stings is of importance due to the lack of understanding of the venom composition, a lack of EBM, and to ensure industry is not highly impacted. Jellyfish warning signs have been erected along the Mediterranean coast in some jellyfish hotspots, yet should be more widely distributed. The promotion of wetsuits and rubber shoes when entering the water in summer months, as well as slow entrance into the sea to be aware of jellyfish should be advertised throughout the summer months. Divers should ensure the correct protocol during resurfacing is carried out with one arm raised above their head to avoid a more severe sting if there is contact with jellyfish. If jellyfish are sighted, a wide berth is advised as their tentacles may extend far from the main body [23]. The use of anti-jellyfish nets should be used as a mitigation tool in highly impacted areas to allow beachgoers to swim in a sting free zone. These nets have led to a 90% sting reduction in the Mediterranean and should be more widely distributed which may prevent the closure of beaches in high risk locations [68].

Med-Jellyrisk have developed an app and website to release morning alarms for the Spanish coast to provide real-time and accurate information to beachgoers on whether there are jellyfish on the beach and the threat of these species with a color coded risk index. The enhanced use of this tool will prevent the currently high number of jellyfish stings along the Mediterranean, estimated at ~65%–75% of injuries on the Spanish coastline [69]. This interactive citizen science tool will enable first aid intervention to be reduced if universally implemented in vulnerable localities and allow the distribution of jellyfish blooms to be more greatly documented to foresee future blooms with the correlation of environmental factors.

It has been established by Bordehore et al. that there is a lack of information distributed by tourist information offices in Spain on the threat of jellyfish stings and possible methods of treatment. There is a reluctance to provide information due to the perception that tourism may decrease due to the fear of being stung. However, with the assignment of these avoidance techniques and development of suitable first aid treatments, this will prevent an impact on the tourist industry, allow the management of jellyfish sting threat, and reduce the number of stings encounters [69].

## 7. Discussion

### 7.1. Envenomation

Humans face a variety of dangers from jellyfish species across European waters. The threat encountered upon a jellyfish sting depends on the species, the anatomical penetration, and geographic location. The sting results in a cutaneous reaction in most cases, and systemic symptoms from a larger envenomation due to a toxic effect of the venom. Cross-reactivity can lead to a severe allergic reaction of angioedema or anaphylaxis. It is recommended that an individual is evaluated for the development of allergies after a severe jellyfish sting, as it may be necessary to carry an epinephrine pen and antihistamines when returning to the sea [10,54]. The prevention of envenomation should be a priority through wearing protective equipment such as wet suits when entering the water and avoidance of areas with jellyfish blooms.

Further research into the life cycles and life histories of all envenoming species would be advantageous to establish what factors are important in the development of nematocysts, toxins and where jellyfish polyps are likely to develop. There is a considerable knowledge deficiency relating to jellyfish life histories. Specific research may lead to better understanding of the development of venomous species and how this can be avoided and treated [6]. The monitoring of biotic and abiotic factors, such as how prey availability and temperature affect the development of nematocysts and the venom. This may lead to an understanding of how to tackle blooms and envenomations as well as recognizing why certain areas have more dangerous species [6,43].

The study of Wiebring et al. [6] has identified how relative frequencies of nematocysts correlate with the available prey species; thus, the nematocysts reflect the diet of the jellyfish [70]. Accordingly nematocysts treatment can be more specifically analyzed in a given area if prey populations are monitored and subsequently lead to nematocyst or toxin-specific treatments for envenomation. Nematocyst type can have a great effect on the envenomation severity due to the tubule length and thus this research would be beneficial.

There is confusion on species causing different sting severities in various locations. DNA sequencing of jellyfish species may explain why reactions vary between areas. Identification of DNA similarities may lead to better understanding of how envenomation treatment is beneficial in a genus or species specific manner, with certain species closely related perhaps sharing favorable methods of first aid treatment. This research established speciation occurred between the “New World” and “Old World” *Aurelia aurita* and thus why in some areas the species is venomous, and innocuous in others [71].

The effect of a changing climate may alter the distribution of species and envenomation ability, therefore, the monitoring of these factors is highly recommended. Variations in nematocyst length and venom potency have been identified outside of European waters in *A. aurita* and *Cassiopea* sp. dependent on location, habitat and environmental conditions. This emphasizes the threat of climate change altering which species threaten human safety. *Cassiopea* sp. have been reported to be venomous or not, depending on the habitat. The individual jellyfish that were discovered all have identical cnidoms thus, the venom composition must be the variation. *Cassiopea* sp. has been found to possess dinoflagellates in their venoms, which may be another factor that alters toxicity between geographic locations [43]. Further research is necessary to identify the factors that lead to the variation between geographic localities, as this vital piece of information is necessary in order to develop the most effective therapy.

The threat of climate change could also facilitate a change in the geographic distribution of species, as well as their physiology and toxicity. The El Niño effect in Florida, which increases the water temperature, led to an increased length of tentacles in *A. aurita* and subsequently increased the ability of envenomation [43,62]. Events such as the findings from the El Niño portray how environmental factors may play a large role in the toxicity of species and why species can vary due to localities they inhabit. This climate change effect could be mirrored in European species, thus, environmental factors should be further investigated as these changes may lead to mild species becoming an increased threat to the public.

The identification of human-mediated activities on jellyfish blooms will enable blooms to be monitored and controlled in relation to overfishing, eutrophication, and construction [72]. Many jellyfish species polyps have been found to favor artificial materials and man-made developments. The development of a protocol to monitor materials used in coastal development will aid in the surveying and avoidance of jellyfish blooms arising in popular areas. Factors such as heavy metals have been proven to affect nematocysts discharge response and the biological activity of crude venom [73]. Therefore, if such factors are polluting certain areas it may explain why there is variability in the severity of the sting.

### 7.2. Treatment

First aid treatment is the primary concern when dealing with jellyfish stings. The process that is carried out to remove tentacles and treatment of the stung area can be crucial to avoid the further release of nematocysts and heightened symptoms through a change in osmotic pressure or mechanical stimulation [25]. Anecdotal treatments are more common than EBM for jellyfish stings due to a lack of understanding of jellyfish venom and patient variability playing a large role in the efficacy of the therapeutic agents. Many treatments that have previously been advised are a counteractant or a placebo. Ultimately much of what is considered a beneficial therapy is unavailable for immediate first aid response such as IV channel blockers. Topical therapy is unable to catch up with the initial severe envenomation as the toxin is injected into the dermis directly, whilst topical treatment takes thirty minutes to reach the same area as the initial sting [16,18].

IV therapy is unavailable at the scene directly after the envenomation unless IV first aid protocol has been set up in the most dangerous areas. Although IV channel blockers are shown to be beneficial for nematocyst inhibition and pain relief with 100% (11 papers) efficacy, there is the threat of disguising a medium to severe sting, and hence, leading to systemic complications remaining undetected and resulting in the danger of life. Complications of delayed kidney and liver failure have been reported after medium envenomation, referred to as delayed jellyfish envenomation syndrome (DJES) [12,29,35]. Deaths within two hours of envenomation are typically resultant from cardiotoxicity due to large doses of venom generating cardiovascular collapse. Cardiotoxicity results from the ionic disturbances with the loss of potassium and increase in sodium and calcium ions leading to the ultimate loss of action potentials, with the venom influencing calcium binding [40,74,75]. Moderate envenomation depresses respiration through the central nervous system minutes to hours after the sting. The deaths that occur between 2 h and 48 h after a lower dose of venom may be due to DJES through cellular or tubular necrosis. The proteolytic enzymes in the venom are believed to cause a hemorrhage through the breakdown of connective tissue around blood vessels and extracellular matrix, injuring the organs. The vascular system seems to be one of the main targets of the venom, similar to snake venom, containing metalloproteins, caseinolytic, and fibrinolytic activities [12,32,33,45]. Understanding aspects of the venom and the microvascular network is important to establish the mechanism that leads to DJES to create appropriate intervention therapies, with further research required to understand if calcium and sodium channel blockers aid cardiotoxicity but mask DJES.

The rate of venom uptake is dose dependent and relative to the peripheral circulation, thus, the victim must be kept calm and immobile to prevent increased blood flow. Peripheral circulation of the venom is mainly governed by muscular contraction of the surrounding envenomed tissue as well as the total injected dose of venom, however PIB is no longer recommended to reduce blood flow as the pressure may discharge remaining nematocysts [10,12,17,27,76].

Freshwater should never be used as a treatment for jellyfish stings as a change in osmotic concentration can trigger nematocyst release further intensifying the initial sting. Sea water has been established to be an effective painkiller and should be used in aqueous treatments, such as baking soda slurry, as an immediate therapy and to wash off tentacles [6,8,10].

A great deal of dispute over the efficacy of treatment is due to the different methods of venom extraction displaying a variation in results. Several studies have identified differences between the biological activities of nematocysts venom compared to tentacle-only extract (TOE) [33,75,77]. These investigations using TOE produce false positive results and highlight problems with research into the testing of treatments.

A new research method, published by Yanagihara et al. assessing the efficacy of topical first aid treatment on cubozoan tentacle firing and venom toxicity presents a new in vitro method of testing. This involves a living sting model composed of human red blood cells suspended in agarose gel, covered by lanolin-rubber sterile porcine intestine as a mock skin and live stinging tentacles to test first aid measures. This avoids ethical issues of experimenting on humans while allowing the measurement of first aid with live tentacle applicant using an artificial and cost-effective model that could be utilized to test other jellyfish species [17].

The research of Cuypers et al. [65] identified TRPV1 receptors to be associated with cnidarian envenomation, which is pH and heat sensitive. Further to this, scyphozoan venoms have been identified to have heightened hemolytic activity at low temperatures and are dramatically reduced upon an increase in pH and temperature [78]. Many anecdotal treatments are light acids in an attempt to relieve pain; however, further investigation into blocking these receptors without the further release of nematocysts would produce an effective pain relief treatment. The investigation into infrequent therapies that have been identified as showing a beneficial response could prove favorable for first aid treatment of pain and deactivation of venom, such as palm oil and lemon juice emulsion which is a common anecdotal therapy in the tropics [79]. Jellyfish envenomation studies should concentrate on genus based or individual species treatment as opposed to a general protocol for a better therapeutic impact, using large sample sizes, double blind tests and species from various areas, as well as the use of a dolorimeter to assess the intensity of pain if focus is on a case by case level.

The presence of first aid stations and response teams along the coastlines of highly affected areas in Europe is recommended, for instance along the Mediterranean coast. Hot showers erected on beaches that distribute water at a continuous temperature of between 42 °C and 45 °C are recommended to suppress pain and to deactivate venom. Baking soda and Stingose should be available along high-risk areas as an immediate response. These three therapies have displayed beneficial outcomes of first aid response for a variety of jellyfish species envenomations and are available at a low cost with no necessary training in the administration of these treatments. Cold packs are another treatment that has displayed a highly beneficial outcome across a number of species. Cold packs in mild envenomation cases are beneficial as there has been no adverse effect reported with this treatment due to the cold resulting in a slow diffusion rate of venom around the body, unlike heat mediated treatments. Despite this, extreme heat has proven more favorable across a number of marine envenomations and is effective on TRPV1 receptors, unlike extreme cold [34,36,80,81,82,83].

The training of lifeguards and education on jellyfish envenomation is recommended, in addition to the distribution of signage to warn public and tourists of how to avoid the dangers of jellyfish, what species are most hazardous and a recommended first aid protocol with post-sting infographics to help establish the best form of species-specific treatment. A forecast of jellyfish sightings is recommended on popular beaches on the coasts of Europe that have a high frequency of jellyfish. At peak times with the presence of blooms, it may be advisable to cordon off beaches or implement the use of anti-jellyfish nets, as well as develop monitoring protocols to detect when and where a bloom may arise. The promotion of citizen science tools will enable jellyfish blooms to be monitored to a better degree, prepared for, and identify the best intervention techniques through the tracking of environmental factors during their occurrence.

## 8. Conclusions

The consensus from reviewed papers is that there is a lack of information on how best to treat jellyfish stings due to limited data of EBM. Many tested jellyfish therapeutics have poor, nonrandomized trials leading to the implementation of anecdotal remedies. First aid treatment is the primary concern when dealing with jellyfish stings, as the process that is carried out to remove the tentacles and treatment of contacted area can be crucial to avoid the further release of nematocysts and heightened symptoms. Pathology of a jellyfish envenomation is venom dose-dependent. With severe envenomation, from a large jellyfish and significant stung surface area, fast and efficient first aid response is vital to avoid an increase in venom load and evade further harm thus preventing a fatal reaction with the more venomous species.

Emergency responders do not have standardized approaches available, thus, protocols must be developed to minimize complications particularly in remote areas. Until further specified investigation is carried out on species-specific treatment, it is recommended to follow the general treatments above and reference to the MED-jellyrisk sting protocol, as well as avoidance of jellyfish blooms at peak times and protection to avoid initial contact with all jellyfish [22].

## Figures and Tables

**Table 1:** List of possible symptoms, location and sting characteristics that have been discussed in reviewed papers.

Species	Sting Severity	Species Frequency	Area	Cutaneous Symptom	Systemic Symptom	Post-Sting Marking	Reference
Scyphozoa							
*Atolla* sp.	Mild	Low	NA	NRF	NRF	NRF	[42]
*Atorella subglobosa*	Mild	Very low	NA	NRF	NRF	NRF	[42]
*Aurelia aurita*	Mild	High	E	Dermatitis Hives Pain Piloerection	NRF	NRF	[9,19,22,34]
*Cassiopea andromeda*	Mild	High	MS NA	NRF	NRF	NRF	[19,22,43]
*Catostylus tagi*	Mild	Low	NA	NRF	NRF	NRF	[21,44]
*Chrysaora hysoscella*	High	Low	CS IS MS NS	Wheals Punctuate erythematous rash	NRF	Multiple punctate linear wheals; Pink macules which coalesce; Discontinuous line of regularly distributed small papules surrounded by erythematous halo	[9,22,28]
*Cotylorhiza tuberculata*	Mild	High	MS NA	NRF	NRF	NRF	[19,22]
*Cyanea capillata*	Severe*	High	MS NA NS	Burning Edema Erythema Irritations Pain Wheals	Abdominal pain Cardiac complications Dizziness Drowsiness Hemolysis of red blood cells Impaired consciousness Organ failure Muscular cramp Nausea Pain Profuse sweating Respiratory distress	Red erythematous stripes; Fine, stippled, linear wheal bordered by narrow flare: circular saw tooth pattern wheal	[7,9,12,23,32,34,40,45,46,47]
*Cyanea lamarckii*	Mild	High	NA NS	NRF	NRF	NRF	[48]
*Deepstaria enigmatica*	Mild	Very low	NA	NRF	NRF	NRF	[49]
*Discomedusa lobata*	Mild	Very low	MS	NRF	NRF	NRF	[19,22]
*Drymonema dalmatinum*	High	Very low	NA	NRF	NRF	NRF	[22]
*Marivagia stella*	Mild	Low	MS	NRF	NRF	NRF	[22]
*Nausithoe* sp.	Mild	Low	NA	NRF	NRF	NRF	[19]
*Paraphyllina ransoni*	Mild	Low	MS NA	NRF	NRF	NRF	[50]
*Pelagia benovici*	Medium	Low	MS	NRF	NRF	NRF	[51]
*Pelagia noctiluca*	High	Very high	MS	Burning Dermatitis Edema Erythema Hives Hyperpig-mentation Lesions Pain Vesicles	Anaphylaxis Dizziness Diarrhea Dyspnea Guillian Bare-Syndrome Hypotension Shock Vomiting	NRF	[9,10,19,22,34,38,39,52,53,54,55,56]
*Periphylla periphylla*	Mild	Low-High	NA	NRF	NRF	NRF	[44]
*Phacellophora camtschatica*	Mild	Low	NA MS	NRF	NRF	NRF	[44,57]
*Phyllorhiza punctata*	Mild	Low	MS	NRF	NRF	NRF	[22]
*Poralia rufescens*	NRF	Low	NS	NRF	NRF	NRF	[44]
*Rhizostoma pulmo (Rhizostoma octopus)*	Mild	High	BS IS NS MS	Erythema Pain	NRF	NRF	[19,22]
*Rhopilema luteum*	Medium	Very low	MS NA	NRF	NRF	NRF	[22]
*Rhopilema nomadica*	Medium	High	MS	Burning pain Erythematous eruptions	Delayed cutaneous reactions Fatigue Fever Muscular aches	NRF	[19,22,34]
*Rhopilema rhopalophora*	NRF	Rare	NA	NRF	NRF	NRF	[21]
*Stephanoscyphus mirablis*	NRF	Rare	NA	NRF	NRF	NRF	[21]
*Stygiomedusa gigantea*	NRF	Rare	NA	NRF	NRF	NRF	[21]
Cubozoa							
*Carybdea marsupialis*	High	High	MS NA	Inflammation Pain	Cardiac complications Irukandji syndrome Muscular cramps Neurological complications	NRF	[7,22,25,34,41]
Hydrozoa							
*Gonionemus vertens*	Medium	High	MS NA NS	Burning pain Edema	Convulsions Disturbed respiration Neuro-psychiatric changes	NRF	[22,58]
*Olindias phosphorica*	Medium	Low	MS NA	NRF	NRF	NRF	[22]
*Physalia physalis*	Severe *	High	MS NA	Atrophy of subcutaneous tissue Blistering Edema Erythema Hyper-pigmentation Inflammation Keloids Lesions Linear plaques pain Long lasting dermal marks Necrosis Pain Pigmentation Pruritus Recurrent rash	Abdominal pain Anaphylaxis Angioedema Coma Confusion Cardiac complications Cold sweats Cyanosis Death Diarrhea Drowsiness Dyspnea Fainting Gastro-intestinal allergies Headache Haemolysis Hysteria Irukandji syndrome Joint pain Muscular spasm Nausea/Vomiting Neurological complications Nervousness Organ failure (kidney/liver) Pallor Parasympath-etic dystopia Reactive arthritis Respiratory complications	Linear line of lesions (like a row of beans); Segmented/banded/crossed skin wheals	[7,8,9,10,22,25,26,28,34,36,59,60,61,62]

Mild: Cutaneous marks, with minimal discomfort which fade after several hours. Medium: Cutaneous reaction, with the threat of some systemic symptoms. High: Cutaneous and systemic symptoms occur more commonly. Severe*: Have been known to rarely result in fatalities with recorded cases of death from systemic complications. E: Europe-wide; MS: Mediterranean Sea; NA: North Atlantic Ocean; CS: Celtic Sea; IS: Irish Sea; NS: North Sea; BS: Black Sea; NRF: no reports found.

**Table 2:** Conclusive effect of treatment on post-sting treatment for commonly reviewed species with number of papers.

Treatment	*A.a.*	*C.h.*	*C.c.*	*P.n.*	*R.p.*	*R.n.*	*C.m.*	*P.p.*
**Acid**			1−	1−				
**Alcohol**			1,1−	1, 1−			1−	1, 3−
**Ammonia**				1+				1−
**Analgesic**								1+
**Anesthetic**				1+				2+
**Anions**				1−				
**Antihistamine**								2+
**Anti-inflammatory**				1+	1+			6+ ,1−
**Antivenin**				1+				1
**Baking soda slurry**		3+	2+	1−				2+, 1
**Bases**				1−				
**Channel blocker**			4+	2+			2+	1+
**Cold pack**		1	2+	2+				4+, 1
**Heat**	1+	1+, 1	2+	1+, 1−		1+	1+	4+, 2−
**Palm oil and Lemon juice**							1+	
**Pressure inducing bandages (PIB)**				1, 1−				
**Sand**								1+
**Sea water**	1+	1+	2+	3+				1+
**Splint**							1+	
**Stingose**			1+				1+	2+
**Thioglycolate**				1−				
**Urine/urea**			1−					
**Vinegar**	1−	1+	1+, 4−	2+, 3−			3+	6+, 1, 6−
**Vitamin C**			1+					
**Vitamin E**				1+				
*A.a.: A. aurita*; *C.h.: C. hyscoscella*; *C.c.: C. capillata*; *P.n.: P. noctiluca*; *R.p.: R. pulmo*; *R.n.: R. nomadica*; *C.m.: C. marsupialis*; *P.p.: P. physalis*.								
Key:	Overall positive effect (+)			Overall no effect		Overall negative effect (−)		

## Abbreviations

The following abbreviations are used in this manuscript:

EBM	evidence-based medicine
WOK	Web of Knowledge
WoRMS	World Register of Marine Species
NRF	no reports found
NA	North Atlantic
E	Europe-wide
MS	Mediterranean Sea
NS	North Sea
CS	Celtic Sea
IS	Irish Sea
BS	Black Sea
Ig	Immunoglobulin
IV	Intravenous
PIB	pressure inducing bandages
*A.a*.	*Aurelia aurita*
*C.h.*	*Chrysaora hysoscella*
*C.c.*	*Cyanea capillata*
*P.n.*	*Pelagia noctiluca*
*R.p.*	*Rhizostoma pulmo*
*R.n.*	*Rhopilema nomadica*
*C.m.*	*Carybdea marsupialis*
*P.p.*	*Physalia physalis*
DJES	delayed jellyfish envenomation syndrome
TOE	tentacle-only extract
